# The care pathway for children with urticaria, angioedema, mastocytosis

**DOI:** 10.1186/s40413-014-0052-x

**Published:** 2015-02-02

**Authors:** Giuliana Ferrante, Valeria Scavone, Maria Concetta Muscia, Emilia Adrignola, Giovanni Corsello, Giovanni Passalacqua, Stefania La Grutta

**Affiliations:** Department of Science for Health Promotion and Mother and Child Care, Università di Palermo, Via del Vespro, 133, 90127 Palermo, Italy; Allergy and Respiratory Diseases, Department of Internal Medicine, IRCCS San Martino, University of Genoa, Genoa, Italy; Institute of Biomedicine and Molecular Immunology IBIM, National Research Council, Palermo, Italy

**Keywords:** Urticaria, Angioedema, Mastocytosis, Skin, Itch, Children, Epidemiology, Diagnosis, Management, Clinical practice

## Abstract

Cutaneous involvement characterized by urticarial lesions with or without angioedema and itch is commonly observed in routine medical practice. The clinical approach may still remain complex in real life, because several diseases may display similar cutaneous manifestations. Urticaria is a common disease, characterized by the sudden appearance of wheals, with/without angioedema. The term Chronic Urticaria (CU) encompasses a group of conditions with different underlying causes and different mechanisms, but sharing the clinical picture of recurring wheals and/or angioedema for at least 6 weeks. Hereditary Angioedema (HAE) is a rare disorder characterized by recurrent episodes of non-pruritic, non-pitting, subcutaneous or submucosal edema affecting the extremities, face, throat, trunk, genitalia, or bowel, that are referred as “attacks”. HAE is an autosomal dominant disease caused by a deficiency of functional C1 inhibitor, due to a mutation in C1-INH gene (serping 1 gene) characterized by the clonal proliferation of mast cells, leading to their accumulation, and possibly mediator release, in one or more organs. In childhood there are two main forms of mastocytosis, the Systemic and the Cutaneous. The clinical features of skin lesions in urticaria, angioedema and mastocytosis may differ depending on the aetiologic factors, and the underlying pathophysiological mechanisms. The diagnostic process, as stepwise approach in routine clinical practice, is here reviewed for CU, HAE and mastocytosis, resulting in an integrated method for improved management of these cutaneous diseases. Taking into account that usually these conditions have also a relevant impact on the quality of life of children, affecting social activities and behavior, the availability of care pathways could be helpful in disentangle the diagnostic issue achieving the most cost-effective ratio.

## Introduction

Cutaneous involvement characterized by urticarial lesions with or without angioedema and itch is commonly observed in routine medical practice, and often this observation poses important diagnostic challenges. In fact, although the diagnostic processes are quite standardized, based on scientific evidences [1], the clinical approach may still remain complex in real life, because several disease may display similar cutaneous manifestations. For instance, itching is useful in confirming the diagnosis of urticaria, but itching is can also be a sign of other systemic diseases, thus requiring additional investigations for an accurate diagnostic process. Therefore, a comprehensive approach to the most common cutaneous allergic diseases occurring in childhood is advised, to achieve an optimal clinical management.

This review describes the clinical features of Urticaria, Angioedema, and Mastocytosis in childhood, including epidemiology. The diagnostic process, as stepwise approach, in routine clinical practice is reviewed for chronic urticaria (CU), hereditary angioedema (HAE) and mastocytosis, resulting in an integrated method for improved management of these cutaneous disease.

### Clinical aspects

#### Urticaria

Urticaria is a common disease, affecting 15-25% of the population with at least one episode during lifetime. In children, urticaria appears to be less common. In the United Kingdom the occurence of all forms of childhood urticaria is around 3,4% [2], while in Germany it has been estimated to be around 4,4% [3] and in Denmark around 5,4% [4]. The prevalence of childhood chronic urticaria is 0,1-0,3% in the United Kingdom [5], while in a Thai study, 13% of children with urticaria were classified as having a chronic form [6].

A clinical classification of urticaria has been recently published in a European Academy of Allergy and Clinical Immunology (EEACI) guideline [7] (Table 1).

**Table 1:** **Classification of urticaria subtypes (Modified From EEACI guideline 2009)**

**Types**	**Subtypes**
Spontaneous urticaria	Acute Spontaneous Urticaria (<6 weeks)
	Chronic Spontaneous Urticaria (>6 weeks)
Physical urticaria	Cold Contact Urticaria
	Delayed Pressure Urticaria
	Heat Contact Urticaria
	Solar Urticaria
	Urticaria Factitia/Dermographic Urticaria
	Vibratory Urticaria/Angioedema
Other urticaria types	Aquagenic Urticaria
	Cholinergic Urticaria
	Contact Urticaria
	Exercise Induced Anaphylaxis/Urticaria

Urticaria is characterized by the sudden appearance of pruritic wheals, with/without angioedema. A wheal consists of three typical features: 1. a central swelling of variable size, almost invariably surrounded by a reflex erythema; 2. itching or, sometimes, burning sensation; 3. a fleeting nature, with the skin returning to its normal appearance, usually within 1–24 h. Urticaria is defined as acute if wheals last less than 6 weeks, and chronic if lasting 6 weeks or more [8]. The term Chronic Urticaria (CU) encompasses a group of conditions with different underlying causes and different mechanisms, but sharing the clinical picture of recurring wheals and/or angioedema for at least 6 weeks [9]. Chronic Spontaneous Urticaria (CSU) is defined as the daily or almost daily occurrence of wheals for more than 6 weeks, after the exclusion of physical urticaria and other urticarial types [10].

CU in children is caused by physical factors in at least 6% of cases. Less often, infections (4%), foods (4%), additives (2,65%), aeroallergens (2,2%) and drugs (1,6%), are trigger factors [4]. Physical Urticaria (PU) is a heterogeneous subgroup of chronic urticaria in which wheals can be induced by various physical stimuli such as cold, heat, pressure, vibration, or sunlight. It is common in young adults [10], whereas in children cold urticaria is idiopathic or secondary to viral infections [11]; in addition some atypical cold urticaria forms have been reported, being either hereditary or acquired [12].

Other urticarial disorders include cholinergic, acquagenic, contact- and exercise-induced urticaria [8,9]. The formers, with typical small wheals (diameter of less than 5 mm), appear within a few minutes after the elevation of body temperature, independently of passive exposure to hot shower, or as a consequence of physical exercise [13,14].

#### Angioedema

There are few epidemiological data about Angioedema in children. In the only published study on children with urticaria, wheals alone were present in 78,4%, angioedema alone in 6,65% and both in 15% [15]. Angioedema is characterized by: 1. a sudden, pronounced swelling of the lower dermis and subcutis; 2. sometimes pain rather than itching; 3. frequent involvement of mucous membranes; 4. slow resolution that can take up to 72 h [7].

Hereditary Angioedema (HAE) is a rare disorder (Orpha number 91378, http://www.orpha.net) characterized by recurrent episodes of non-pruritic, non-pitting, subcutaneous or submucosal edema affecting the extremities, face, throat (tongue, larynx, and lips), trunk, genitalia, or bowel, that are referred as “attacks”. Classically edema develops slowly, over a period of up to 36 hours and completely resolve spontaneously within 4 days. Swelling is usually self-limited and painless when affecting the skin, whereas abdominal attacks with diarrhea, vomiting, and pseudo-obstructive syndrome are painful, resulting sometimes in unnecessary surgery. All attacks localized over the shoulders must be considered severe and potentially life-threatening [15]. In some patients a mean interval of 8.3 hours between onset and maximum development of laryngeal edema was reported [16]. In the United States a HAE incidence rate of 1:10,000-50,000 people has been reported, and a recent study underlined the importance of diagnosis and appropriate treatment, as the mortality of HAE patients who had not been diagnosed was 29% compared to 3% in those who had been diagnosed [17]. Moreover, a study highlighted the high frequency of *de novo* mutations and exon deletions in the *C*_*1*_*inhibitor* gene of patients with angioedema without family history, suggesting important and incoming acquisitions in the genetic epidemiology of the disease [18].

HAE is an autosomal dominant disease caused by a deficiency of functional C1 inhibitor (C1-INH), due to a mutation in C1-INH gene (serping 1 gene) mapped to chromosome 11(11q12-q 13.1). Bradykinin is the main mediator implicated in edema [19]. Although more than 200 mutations of serping 1 gene have been linked to the clinical HAE manifestations, only two account for the majority of cases [20]. An estimated 85% of patients have Type-1 HAE (Table 2).

**Table 2:** **HAE Classification**

HAE 1	Low production of functionally active C1-INH
HAE 2	Normal or elevated levels of C1-INH, but with functional impairment of the protein
HAE3 (irrelevant in children and adolescent)	Mutations in the coagulation factor XII. No abnormalities in C1-INH level or function

#### Mastocytosis

Mastocytosis is a heterogeneous disorder characterized by the clonal proliferation of mast cells (MCs), leading to their accumulation, and possibly mediator release, in one or more organs [21]. In childhood there are two main forms of mastocytosis, the Systemic (SM) and the Cutaneous (CM), this latter including three major variants: maculopapular cutaneous mastocytosis (MPCM), diffuse cutaneous mastocytosis (DCM), and solitary mastocytoma. The classification has been recently reviewed by the World Health Organization (WHO) [22].

In Spain a prevalence of 5.4 cases per 1000 pediatric dermatology patients was found, whereas in Mexico the condition was seen in 1:500 first-time pediatric dermatology patients. Mastocytosis occurs in all ethnic groups and may appear at any age. CM is more common in children; a second smaller peak of incidence is seen in adults in the third to fourth decade [23].

MPCM (urticaria pigmentosa) is the most common clinical variant in which fixed, reddish brown lesions occurring as maculo-papules, plaques, nodules or blisters are found. These lesions urticate in response to physical irritation (Darier’s sign). Urticaria Pigmentosa (UP) lesions tend to be larger, better delineated, and more hyperpigmented in children, as compared to adults, who tend to have numerous small lesions that coalesce to form mottled areas. The trunk and thigh are more commonly involved with sparing of face, palms and soles [21].

DCM is a rare variant of childhood mastocytosis that appears as diffuse infiltrative yellow-orange xanthogranuloma-like subcutaneous nodules, or as a widespread urticarial eruption with bullae and redness. The clinical course of DCM is more severe than that of mastocytoma and MPCM and can even be life-threatening, due to hypovolemic shock, mast cell leukemia, gastrointestinal hemorrhage, and cachexia [24].

### The stepwise diagnostic approach

#### Chronic urticaria

A step by step diagnostic approach or chronic urticaria is recommended, starting from the patient history and physical examination, in order to better address the subsequent tests, some of which are performed to exclude severe systemic diseases (Table 3).

**Table 3:** **First and second tools of chronic urticaria**

**Tools**	**Chronic urticaria**
**First line**	•**Relevant questions:**
	Q1. time of onset of disease
	Q2. frequency and duration of wheals
	Q3. diurnal variation
	Q4. shape, size, and distribution of wheals
	Q5. associated angioedema
	Q6. associated subjective symptoms of lesion
	Q7. family and personal history regarding urticarial atopy
	Q8. previous or current allergies, infections, internal diseases, or other possible causes
	Q9. psychosomatic and psychiatric diseases
	Q10. surgical implantations and events during surgery
	Q11. gastric/intestinal problems (stool, flatulence)
	Q12. induction by physical agents or exercise
	Q13. use of drugs (nsaids, injections, immunizations, hormones, laxatives, suppositories, ear and eye drops, and alternative remedies)
	Q14. observed correlation to food
	Q15. relationship to the menstrual cycle
	Q16. smoking habits
	Q17. type of work
	Q18. hobbies
	Q19. stress (eustress and distress)
	Q20. occurrence in relation to weekends, holidays, and foreign travel
	Q21. quality of life related to urticaria and emotional impact
	Q22. previous therapy and response to therapy
	•**Physical examination**
	•**According to symptoms:** Complete blood count, liver and renal function tests, C-reactive protein levels and sedimentation rate urinalysis
**Second line**	**Vasculitis:** Ig, antinuclear factor, immune complex, skin biopsy
	**Infections:** Serologic studies for: HCV,HBV; HAV, EBV, Mycoplasma Pneumoniae. Cultures, fecal antigen of Helycobacter Pylori or Urea breath test.
	**Allergic:** IgE, skin tests, eosinophil count, challenge, elimination diet, tryptase.
	**Hereditary angioedema:** C3,C4, C1 esterase inhibitor.
	**Physical:** Methacolin test, running test, ice cube test, UV and visible light exposure, dermographism, pressure test, warm water immersion test.
	**Autoimmune chronic urticaria:** Autologous serum skin test (ASST), basophil histamine release test (BHRT).
	**Other:** FT3,FT4, TSH, thyroid autoantibodies, celiac screening.

#### First line

Clinical history, including all possible eliciting factors and significant aspects of the origin of urticaria, is the most important issue for a proper diagnostic process. Some of the most frequently asked questions are reported in Table 4. Several scoring systems have been proposed using scales from 0–3 or up to 10 points. A unified and simple scoring system, the Urticaria Activity Score (UAS), was recently validated for the evaluation of disease activity by patients and their treating physicians. As urticarial symptoms frequently change in intensity, overall disease activity is best measured by advising patients to document a 24-hour based self-evaluation score for consecutive days. In CU patients the UAS score of 7 consecutive days should be used to assess the disease activity and the treatment efficacy (Table 4) [7]. Clinical symptoms must determine which laboratory tests could be performed e.g., complete blood count, liver and renal function tests, C-reactive protein levels, sedimentation rate and urinalysis [1].

**Table 4:** **Assessment of disease activity patients with urticaria**

**Score**	**Wheals**	**Pruritus**
0	None	None
1	Mild (<20/24 h)	Mild
2	Moderate (20-50/24 h)	Moderate
3	Intense (>50/24 h)	Intense

Sum of score: 0–6.

(Modified From EEACI guideline 2009).

#### Second line

Additional investigations are suggested in the diagnostic approach to CU and CSU. Although allergy is a rare cause of CSU an allergy screening test should be considered in CSU patients with a personal history suggestive of allergic diseases. Chronic persistent bacterial, viral parasitic and fungal infections that may trigger urticarial symptoms in patients with CSU should be considered, only according to the patient’s history [7]. Total immunoglobulin count and antinuclear factor should be considered in patients with single hives that lasts >24 hours, to rule out vasculitis whereas biopsy is rarely necessary (patients with one or more of the following features: individual painful lesions with purpuric or petechial characteristics that last >24 hours, elevated C-reactive protein levels and/or systemic symptoms such as fever and arthralgias, unresponsiveness to appropriate doses of antihistamines) [8,25]. In Physical Urticaria the routine diagnosis is mainly aimed at the identification of the subtype of the urticaria through the appropriate physical stimulation tests and to the determination of trigger thresholds [7].

Since autoimmune diseases, like celiac or thyroid disease are commonly reported in children with CU, laboratory tests for these diseases should be done [10]. Recent publications have shown 30-50% of patients with CSU produce an immunoglobulin IgG type autoantibody against either the high affinity receptor FcERIα or IgE. However, the utility of the Autologous Serum Skin Test (ASST) remains unclear in identifying a distinct subgroup of patients with CU and in predicting natural history and response to treatment. So, current evidence does not support routine performance of this test in patients with CU [26].

**Figure 1:**
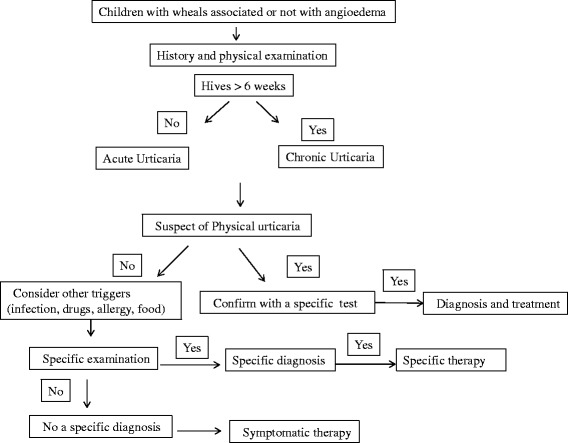
**The diagnostic pathway of Urticaria.** Although allergy is a rare cause of CSU an allergy screening test should be considered in CSU patients with intermittent symptoms and a suggestive history. Chronic persistent bacterial, viral parasitic and fungal infections that may trigger urticarial symptoms in patients with CSU should be considered, only according to the patient’s history [7]. Total immunoglobulin count, antinuclear factor and skin biopsy should be considered in patients with single hives that lasts >24 hours, to rule out vasculitis or Schnitzler syndrome [8]. In Physical Urticaria the routine diagnosis is mainly aimed at the identification of the subtype of the urticaria through the appropriate physical stimulation tests and to the determination of trigger thresholds [7]. Since CU in children may be associated with autoimmune diseases, like celiac or thyroid disease, some Authors suggest to screen for them [9]. 30-50% of patients with CSU produce an immunoglobulin IgG type autoantibody against either the high affinity receptor FcERIα or IgE. However, the utility of the Autologous Serum Skin Test (ASST) remains unclear in identifying a distinct subgroup of patients with CU and in predicting natural history and response to treatment. So, current evidence does not support routine performance of this test in patients with CU [26]. Some other conditions should be considered in the diagnostic process mainly when an immunocomplex-induced disease is suspected. These include serum sickness or autoimmune diseases. When isolated angioedema is present, the assessment of C4 and C1 esterase inhibitor levels should be performed [8].

Some other conditions should be considered in the diagnostic process mainly when an immunocomplex-induced disease is suspected. These include serum sickness or autoimmune diseases (Figure 1). When isolated angioedema is present, the assessment of C4 and C1 esterase inhibitor levels should be performed [8].

#### Hereditary angioedema (HAE)

**Figure 2:**
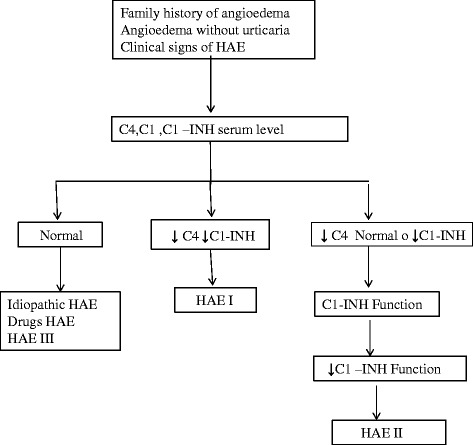
**The diagnostic pathway of Hereditary Angioedema (HAE).** According to the 2010 International Consensus algorithm for diagnosis, therapy and management of HAE, the diagnosis should be confirmed by measuring serum complement factor 4 (C4) and serum C1-inhibitor protein and its functional levels [27].

According to the 2010 International Consensus algorithm for diagnosis, therapy and management of HAE, the diagnosis should be confirmed by measuring serum complement factor 4 (C4) and serum C1-inhibitor protein and its functional levels [27]. Therefore, according to the above section, the diagnostic pathway is showed as *first line* and *second line* tools (Table 5, Figure 2).

**Table 5:** **First and second line diagnostic tools of hereditary angioedema**

**Tools**	**Hereditary angioedema**
**First line**	**Clinical presentation:** recurrent angioedema or abdominal pain in the absence of hives, without urticaria
	**Family history**
	**Laboratory:** C4 and C1 inhibitor serum levels
**Second line**	**Genetic analysis:** mutations in C1-INH gene (Serping1 gene), mapped to chromosome 11 (11q12-q13.1)

#### Mastocytosis

Mastocytosis is essentially suspected on clinical ground, (including identification of UP lesions and recognition of compatible MC mediator-related symptoms). Because mastocytosis is due to a clonal proliferation of MCs, the diagnosis is established by detection of MCs with abnormal morphologic features and immunophenotypes in the bone marrow or other extracutaneous organs and investigating specific mutations in its growth factor (c-KIT) receptor [21].

**Figure 3:**
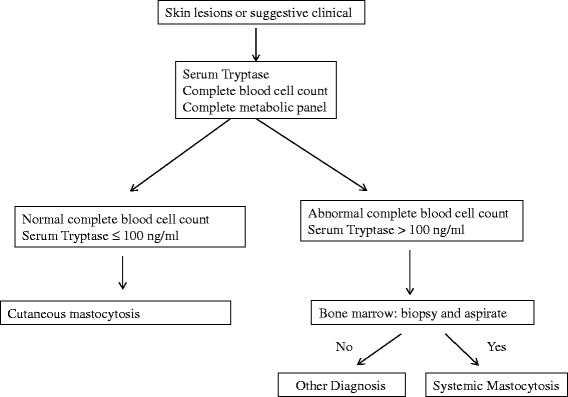
**The diagnostic pathway of Mastocytosis.** Mastocytosis is essentially suspected on clinical ground. Because mastocytosis is due to a clonal proliferation of MCs, the diagnosis is established by detection of MCs with abnormal morphologic features and immunophenotypes in the bone marrow or other extracutaneous organs and investigating specific mutations in its growth factor (c-KIT) receptor [21]. The SCORMA Index provides a standardized information on the severity of CM, with no burden for patients, which is particularly important in children. The simultaneous assessment of SCORMA Index and tryptase level is essential in all cases of pediatric mastocytosis [23].

The SCORMA (SCORing Mastocytosis) Index provides a standardized information on the severity of CM. It is a clinical scoring system based on a semi-quantitative analysis of extent, intensity and subjective complains, with no burden for patients, which is particularly important in children. The simultaneous assessment of SCORMA Index and tryptase level is essential in all cases of pediatric mastocytosis [23]. In Table 6 and Figure 3 the *first line* and *second line* of diagnostic tools and the diagnostic pathway process are respectively showed (Table 6, Figure 3). In some patients with increased tryptase level but not cutaneous signs of mastocytosis levels of other mast cell mediators, such as urinary histamine metabolites (like methylhistamine, NMH or methylimidazole acetic acid, MIMA) and prostaglandin D2, may be increased [28]. In childhood mastocytosis, measurement of the urinary NMH level may be useful at the time of diagnosis high levels of NMH suggesting more severe and extensive disease and possible systemic involvement [29].

**Table 6:** **The first and the second line diagnostic tools of mastocytosis**

**Tools**	**Mastocytosis**
**First line**	Complete blood cell count
	Complete metabolic panel
	Serum Tryptase level
**Second line**	Abdominal ultrasound
	Thoracic X ray
	Bone marrow biopsy and aspirate

### The stepwise management

#### Urticaria

The identification and elimination of eliciting factors is the mainstay approach for urticaria treatment, also considered the special issue of the avoidance of physical stimuli for the treatment of physical urticaria (PU). Since the CSU is often reported to be associated with a variety of inflammatory or infectious diseases, the proper treatment for these underlying conditions should be advised. Although, IgE-mediated food allergy is rarely the underlying cause of chronic spontaneous urticaria, after a confirmed diagnostic tests, the specific food allergens need to be omitted as far as possible [30].

#### First line

In agreement with the most recent international guidelines, non-sedating second generation antihistamines are recommended as first line symptomatic treatment. These drugs have shown to be effective in numerous randomized placebo-controlled clinical trials. First generation antihistamines are equally effective, but they have pronounced anticholinergic and sedative effects which last longer than 12 h, whereas the antipruritic effects last only 4–6 h. In addition, first generation antihistamines can interfere with rapid eye movement sleep, and impact on learning and performance. Thus, the use of first generation antihistamines is discouraged [31].

#### Second line

The second level of therapy includes increasing the dosage of non-sedating second-generation antihistamines. Their mode of action offers the best risk to benefit outcome, in comparison to alternative treatments. At the III level, guidelines recommend to change the non-sedating antihistamine used in level II or to add antileukotriens, as well as a short course of corticosteroids (3 days). At level IV, different treatment options are suggested. H2 antihistamines in combination with H1 antihistamines have a low evidence-based support, whereas more data are available for dapsone and cyclosporine A. However the most effective drug at level IV treatment is Omalizumab [31].

Omalizumab diminished clinical symptoms and signs of chronic idiopathic urticaria in patients who had remained symptomatic despite the use of approved doses of H1-antihistamines. Histamine release from cutaneous mast cells has long been associated with the pathogenesis of urticaria, whereas in patients with chronic idiopathic urticaria, basophils and IgE may also play an important role. Omalizumab, reduces the levels of free IgE and the high-affinity receptor for the Fc region of IgE (FcεRI), both of which are essential in mast-cell and basophil activation. Studies have shown that omalizumab may suppress allergen-mediated skin reactions through its reduction of FcεRI function in basophils and mast cells [32] (Table 7). A study from Maurer et al. found that omalizumab administered as three doses of 75 mg, 150 mg or 300 mg at 4-week intervals significantly reduced symptoms, as compared with placebo, in patients with chronic idiopathic urticaria who remained symptomatic despite the use of approved doses of H1-antihistamines. Clinically meaningful effects were seen for patients receiving either 150 mg or 300 mg of omalizumab in the change from baseline in the weekly itch-severity score (primary end point) and all secondary end points at week 12, with the exception of the proportion of angioedema-free days during week 4 through week 12 in the group receiving 150 mg of the drug [32]. The therapeutic utility of omalizumab for refractory CU is associated with a relatively low rate of clinically significant adverse effects. Hence omalizumab, which use has been approved by the FDA at both 150 mg and 300 mg doses in CU patients unresponsive to H1 antagonists 12 years of age and older, is an alternative therapy to consider if the cost-benefit ratio is favorable and if this approach is consistent with patients’ preferences [11,33].

**Table 7:** **First and second line therapy**

**Chronic urticaria therapy**
**I level therapy:**
• Elimination of possible eliciting factors
• Non-sedating second generation antihistamines (nsAH)
***If symptoms persist after 2 weeks***
**II level:** Updosing of nsAH(X four times)
***If symptoms persist after 4 weeks***
**III level:** Add Leukotriene antagonist or change nsAH
***If symptoms persist after 1–4 weeks***
**IV level:** Add ciclosporin, H2 Antistamine, Dapsone, Omalizumab
**During exacerbation: systemic steroid for 3–7 days in all levels**

Overall, the disease activity should be evaluated at regular intervals, in order to reduce the therapy. After three months of complete response the intensity treatment might be reduced [31].

#### Hereditary angioedema (HAE)

The main treatment of HAE is represented by the control of acute attacks, as well as short-term and long-term prophylaxys [34]. Short-term prophylaxis is intended to protect against an angioedema attack for an important event or for a known trigger. Triggers include dental procedures, surgical procedures, and stressful life events (including in family members, exam times, etc.). Long-term prophylaxis is used to prevent attacks and is indicated for patients with frequent (more than 24 days per year with angioedema symptoms even if mild, or more than 12 severe attacks per year) or severe attacks, past laryngeal attacks, excessive loss of work or school, significant anxiety, and poor quality of life [30]. The drug of choice for children is tranexamic acid. Due to safety concerns, androgen therapy for long-term prophylaxis in children is not usually recommended [27,34].

The first-line therapy for HAE attacks in most countries includes replacement therapy with C1-INH concentrate [35]. Berinert (CSL Behring, Marburg, Germany) is a highly purified, pasteurized and lyophilized plasma-derived C1-INH concentrate that has been approved in more than 30 countries including United States (2009) and Canada (2010) [36]. The largest randomized, double-blind, prospective, placebo controlled study, called International Multicenter Prospective Angioedema C1-inhibitor Trial (I.M.P.A.C.T), confirmed the efficacy and safety of this. The I.M.P.A.C.T.1 trial demonstrated that 20 U/kg C1-INH concentrate is effective in treating acute abdominal and facial HAE attacks [37]. I.M.P.A.C.T.2 was an open-label extension study of I.M.P.A.C.T.1 to evaluate the safety and efficacy of long-term treatment with C1-INH concentrate for successive HAE attacks at anybody location [36]. New drugs such as icatibant (a bradykinin B2-receptor antagonist) and ecallantide (a plasma kallikrein inhibitor) have been developed in recent years. These drugs are not yet available in all countries and are not yet licensed for children. Ecallantide has received a box- warning in the labeling, stating that the drug should not be used as home therapy, because of possible hypersensitivity reactions including anaphylaxis [38]. Studies in children and adolescents are required [37].

Among the available new treatment modalities for long-term prophylaxis, plasma-derived C1-esterase inhibitor (pdC1-INH) concentrates represent an alternative treatment for prevention of HAE attacks. Experimental results showed that outcomes with pd C1 INH treatment of HAE in pediatric patients are comparable to adults [39].

The Food and Drug Administration (FDA) has approved the chronic prophylactic use of nano-filtered C1-inhibitor (nfC1-INH) based on limited data, and it will be important that physicians monitor adverse events carefully. Thus far, it appears that the chronic use of C1-INH is safe and effective [27]. Recent studies concluded that in children nfC1 INH was well tolerated, provided relief from symptoms of hereditary angioedema attacks, and reduced the rate of attacks [40].

The recent literature showed that patients with frequent attacks may benefit from long term prophylaxis. A study evaluated the safety and prophylactic effect of weekly administrations of recombinant C1 INH (rC1 INH). Weekly administrations of 50 U/Kg rhC1INH appeared to reduce the frequency of HAE attacks and were safe and well tolerated [41].

Fresh Frozen Plasma (FFP) is often used for treatment of HAE attacks and for short term prophylaxis. However, the use of FFP should be reserved when C1-INH, icatibant or ecallantide are not available. The appropriate use of disease-specific treatments for HAE improve patients' quality of life and reduce HAE-associated morbidity and mortality. However, many of the newer treatments represent financial challenges due to high costs, which are not always balanced by the reduction of other medical expenses [27].

#### Mastocytosis

Usually, isolated CM needs no treatment or action to be taken. CM with cosmetic complaints should be treated by a topical therapy only in children older than 2 years. When CM is confined to about 10% of body surface, occlusive dressing is used. When the extension is larger, (more than 10%) the occlusive dressing is optional, and usually the corticosteroid cream is diluted [42].

In CM with complaints of itch, redness and swelling, avoiding foods with a possible triggering role has to be suggested. Finally, also a systemic therapy with H1-and H2-blocker (and oral sodium cromoglycate) may be useful [43].

DCM is self-limiting, and the need for a treatment is questionable. If DCM causes significant distress, leads to frequent hospitalizations, or is life-threatening, treatment with imatinib is a good option. Imatinib mesylate, a type II kinase inhibitor, inhibits cell proliferation and induces apoptosis by binding the adenosine triphosphate pocket and the adjacent site of the kinase. The nonselectivity of imatinib also explains side effects such as cardiomyopathy and reduced growth velocity, the latter being a relevant side effect in children. Other side effects include nausea, vomiting, diarrhea, increased liver enzymes, hypophosphatemia, edema, skin rash, granulocytopenia, anemia, and thrombocytopenia, but these are rare in children. A dose of 200 mg/m^2^/day have been used in children, and reducing the dosage as soon as sustained healing of the skin lesions is noted is sufficient to obtain remission. Monitoring of peripheral blood, liver function, cardiotoxicity, and growth velocity is necessary [24].

## Conclusion

The clinical features of skin lesions in urticaria, angioedema and mastocytosis may differ depending on the aetiologic factors, and the underlying pathophysiological mechanisms. For this reason, in routine clinical practice itching and cutaneous lesions generally represent a heavy challenge for the pediatrician in terms of scientific knowledge and use of proper diagnostic procedures, taking into account that usually chronic urticaria have also a relevant impact on the quality of life of children, affecting social activities and behavior. Hence, the availability of care pathways for urticaria, angioedema and mastocytosis could be helpful in disentangle the diagnostic issue achieving the most cost-effective ratio.

## Abbreviations

CU: Chronic urticaria
HAE: ereditary angioedema
EAACI: European Academy of Allergy and Clinical Immunology
CSU: Chronic spontaneous urticaria
PU: Physical Urticaria
C1-INH: C1 inhibitor
MCs: Mast cells
SM: Systemic mastocytosis
CM: Cutaneous mastocytosis
MPCM: Maculopapular cutaneous mastocytosis
DCM: Diffuse cutaneous mastocytosis
WHO: World Health Organization
UP: Urticaria pigmentosa
UAS: Urticaria Activity Score
ASST: Autologous Serum Skin Test
BHRT: Basophil histamine release test
I.M.P.A.C.T: International Multicenter Prospective Angioedema C1-inhibitor Trial
pdC1-INH: plasma-derived C1-esterase inhibitor
nfC1-INH: nano-filtered C1-inhibitor
rC1 INH: recombinant C1 INH
FFP: Fresh frozen plasma
